# Genome-Wide Characterization of the *HaALS* Gene Family Reveals Its Potential Roles in Imazethapyr Tolerance in Sunflower (*Helianthus annuus* L.)

**DOI:** 10.3390/plants15142113

**Published:** 2026-07-08

**Authors:** Pengyuan Xie, Jing Wang, Botong Tong, Chengqian Di, Fei Zhou, Wenjun Wang

**Affiliations:** Institute of Industrial Crops, Heilongjiang Academy of Agricultural Sciences, Harbin 150086, China; xiepengyuan@haas.cn (P.X.); wangjing961@163.com (J.W.); tbotong@126.com (B.T.); dichengqian@haas.cn (C.D.)

**Keywords:** *Helianthus annuus*, acetolactate synthase, *HaALS* gene family, imazethapyr, herbicide tolerance

## Abstract

Acetolactate synthase (ALS; EC 2.2.1.6) catalyzes the first committed step in branched-chain amino acid (BCAA) biosynthesis and is the molecular target of multiple herbicide classes, including the imidazolinones. Here, we performed a genome-wide characterization of the HaALS gene family in sunflower (*Helianthus annuus* L.) and investigated genotype-dependent transcriptional responses to imazethapyr. A total of 11 HaALS genes were identified and classified into three phylogenetic clades (Groups A–C). All HaALS proteins contained the conserved TPP_enzyme domains (TPP_enzyme_N, TPP_enzyme_M, and TPP_enzyme_C), and purifying selection (Ka/Ks < 1) indicated strong evolutionary constraint on their core enzymatic function. Promoter analyses revealed abundant cis-regulatory elements associated with diverse stress and signaling inputs, supporting regulatory potential for herbicide-triggered transcriptional modulation. qRT-PCR analysis following imazethapyr application (0, 24, and 48 h) showed pronounced genotype-dependent expression reprogramming between the susceptible (S) and resistant (R) cultivars. In the R genotype, multiple HaALS members were strongly induced after treatment; specifically, *HaALS4* reached a ~6-fold increase at 24 h and a >10-fold increase at 48 h, and *HaALS11* increased to ~6–7-fold at 24 h while remaining above the baseline at 48 h; several additional paralogs exhibited intermediate induction (~3–8-fold by 48 h). In contrast, the S genotype showed limited changes (typically ~0.8–2-fold). Collectively, these findings define the evolutionary features of the sunflower *HaALS* family and identify herbicide-responsive paralogs that may contribute to imidazolinone tolerance, providing candidates for functional validation and molecular breeding.

## 1. Introduction

Herbicide-driven selection is one of the strongest evolutionary forces shaping the weed populations in agricultural ecosystems [1]. Among the herbicide action targets, the acetolactate synthase (ALS, also known as acetohydroxy acid synthase) is particularly important because ALS represents the key enzymatic step in the biosynthesis of branched-chain amino acids (BCAAs, including valine, leucine, and isoleucine) and is the site of action for multiple herbicide classes [2]. Therefore, ALS inhibitors are widely used in weed control programs, and continuous exposure to these herbicides leads to the rapid emergence of herbicide resistance [3]. The most extensively studied mechanism at present is target resistance, that is, amino acid substitutions in ALS reduce the efficiency of herbicide binding and protect the enzyme’s function under herbicide pressure [4]. In addition to target mutations, more and more evidence indicates that changes in gene copy number, expression level, and regulatory networks also affect the resistance phenotype, especially through dosage/abundance effects and broader stress-response pathways that modulate effective target exposure [5].

In the plant genomes with gene duplication, the ALS-mediated resistance complexity is exacerbated [6]. The presence of multiple ALS paralogous genes complicates molecular diagnosis and affects the evolutionary dynamics of resistance alleles [7]. For instance, multiple ALS copies may dilute the contribution of any single resistance-conferring allele, while also enabling functional compensation among paralogous genes through regulatory divergence and dosage effects [8]. Therefore, characterizing the ALS gene pool and its expression profile under relevant physiological conditions is crucial for understanding the molecular basis of adaptive traits [9]. Besides herbicide resistance, ALS activity directly regulates the availability of amino acids, and the availability of amino acids is closely related to carbon/nitrogen metabolism and overall stress physiology [10]. This metabolic centrality indicates that *ALS* genes may be involved in broader stress adaptation processes, rather than being limited to herbicide sensitivity alone [11]. ALS-inhibiting herbicides include imidazolinones, sulfonylureas, triazolopyrimidines, pyrimidinyl(thio)benzoates, sulfonanilides, and sulfonylamino-carbonyl-triazolinones [7]. These herbicides collectively form the cornerstone of chemical weed control [3]. Among them, imidazolinone herbicides such as imazethapyr and imazamox (Group 2) are widely used due to their broad-spectrum activity and good selectivity for crops in resistant varieties [12]. At the population level, excessive reliance on *ALS* inhibitors has repeatedly led to the rapid evolution of resistance; at the molecular level, the repeated substitution of ALS conserved sites (such as Ala122, Pro197, Ala205, Asp376, Trp574, Ser653, Gly654; standardized numbering) can reduce the binding ability of the inhibitor and generate cross-resistance patterns that vary depending on the herbicide’s chemical properties and allele background [13]. Importantly, resistance outcomes are often influenced by genetic background: gene family size, allelic heterogeneity, and transcriptional regulation can affect the effective target dose and metabolic buffering capacity under herbicide stress [14]. These factors prompt us to perform family-based characterization of the ALS genes in the crop genome and perform genotype-based expression analysis after herbicide exposure [3].

Although the ALS enzyme is functionally important, there are still knowledge gaps regarding the evolution of the ALS gene family in major crops and the regulation of herbicide response [3]. Although in-depth studies on ALS have been conducted from the perspective of target resistance in weeds and model systems [2], systematic studies on the composition, duplication history, and regulatory potential of the ALS gene family in cultivated species are still limited [15]. Moreover, herbicide response often exhibits genotype dependence: within the same species, different genetic backgrounds can exhibit different transcriptional and physiological outcomes under the same herbicide treatment, reflecting the differences in regulatory networks and metabolic plasticity [4]. This genotype-specific pattern is particularly important for understanding how “susceptible” and “resistant” backgrounds reconfigure gene regulation under ALS inhibition, as well as for improving molecular diagnostic and management strategies [16].

Sunflower (*Helianthus annuus* L.) is one of the most important oil crops in the world and is a key component of herbicide-based weed management programs [17]. The development and promotion of imidazolinone-tolerant sunflower varieties have made it possible to effectively control problematic weeds post-emergence using imidazolinone ALS inhibitors, improving weed control effectiveness and production stability [12]. However, the promotion of ALS inhibitor technology has also increased the selection pressure on weed and crop-associated natural populations, highlighting the importance of understanding the organization and regulation of the ALS gene family in sunflowers [4].

This study systematically identified and characterized the ALS (*HaALS*) gene family in sunflowers and examined its transcriptional response to imidazolinone herbicide exposure [18]. We conducted a genome-wide investigation to determine the members of the *HaALS* family and predict their physical and chemical properties [16]. Based on evolutionary and structural evidence, we reconstructed the phylogenetic relationship and examined the conserved domains and gene structure to elucidate the diversification of *HaALS* paralogous genes [17]. Additionally, we analyzed the cis-regulatory elements in the *HaALS* promoter to infer potential regulatory inputs related to herbicide-induced transcriptional regulation [17]. In order to link the family structure and promoter characteristics with the functional output under herbicide challenges, we used quantitative real-time- polymerase chain reaction (qRT-PCR) to assess the expression of *HaALS* in two sunflower varieties with different tolerance to imidazolinone herbicides: susceptible genotype (S) and resistant genotype (R). At the four-leaf stage, an imidazolinone ALS inhibitor was applied to the plants, and samples were collected from the fully expanded upper leaves at 0, 24, and 48 h after application. This design enabled the assessment of transcriptional dynamics from early to late stages under field-related ALS inhibitors and direct comparison of genotype-specific regulatory responses [4]. By integrating genome-wide family characterization with genotype-resolved expression profiling under imazethapyr treatment, this study provides a framework for understanding HaALS evolution and herbicide-responsive regulation in sunflower, and nominates candidate paralogs for functional validation and molecular breeding [16].

## 2. Results

### 2.1. Identification and Physicochemical Properties of Members of the Sunflower Acetyl-L-Lactate Synthase (HaALS) Gene Family

Using the protein sequences of known acetyl-L-lactate synthases (ALS) from Arabidopsis as query sequences, a genome-wide search was conducted in the sunflower genome. Combined with Pfam and SMART domain validation, 11 members of the *HaALS* gene family were identified and named *HaALS*1 to *HaALS*11 in order of their chromosomal location. The physicochemical properties of these encoded proteins were predicted using the ExPASy ProtParam tool (Table 1). The number of amino acids ranged from 1452 (*HaALS*7) to 1968 (*HaALS*8), and the molecular weight (Mw) ranged from 119,218.6 daltons (*HaALS*7) to 162,185.3 daltons (*HaALS*8). The theoretical isoelectric points (pIs) were acidic and showed a narrow distribution, ranging from 4.92 (*HaALS*8, *HaALS*6) to 5.00 (*HaALS*7). The instability indices ranged from 33.76 (*HaALS*7) to 49.04 (*HaALS*10), with most members having values below 40, indicating that they are relatively stable in vitro. The aliphaticity index ranged from 23.02 (*HaALS*8) to 27.12 (*HaALS*11). The total average hydrophobicity (GRAVY) values were all positive, ranging from 0.672 (*HaALS*7) to 0.809 (*HaALS*3), indicating that *HaALS* proteins are generally hydrophobic, which is consistent with the membrane-associated or lipid-interacting functions of ALS. Table 1 lists the gene identifiers and summary statistics (maximum, minimum, and mean) for each parameter. The subcellular localization prediction results show that the 11 *HaALS* proteins are mainly distributed in the chloroplast and cytoplasm: among them, *HaALS*2/4/5/7/10 were predicted to be located in the cytoplasm, while *HaALS*1/3/6/8/9/11 were mainly located in the chloroplast.

### 2.2. Chromosomal Localization of HaALS Genes

**Figure 1:**
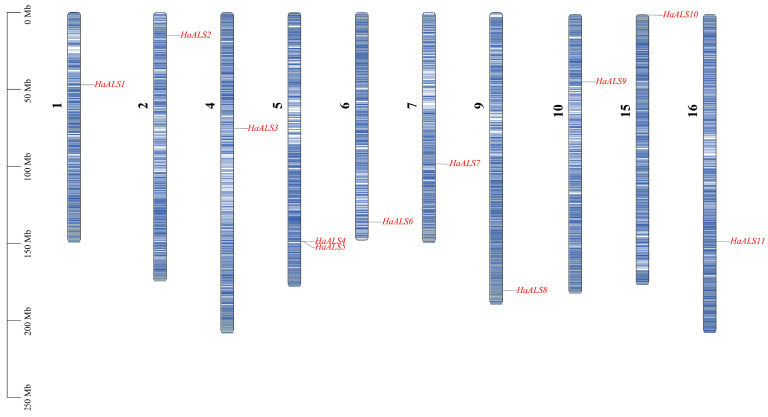
Chromosomal distribution of members of the *HaALS* gene family in sunflower. The physical positions of 11 *HaALS* genes (*HaALS1–HaALS11*) were mapped onto the 17 sunflower chromosomes based on genome annotation data. Each chromosome is shown as a vertical bar with megabase (Mb) scale. Colored markers indicate the chromosomal location of individual *HaALS* genes. Inner tracks display local gene density along each chromosome, with warmer colors or higher histogram peaks representing regions of greater gene density. Eleven *HaALS* genes were unevenly distributed across 10 chromosomes, with no members detected on chromosomes 3, 8, 11, 12, 13, 14, or 17. A tandem cluster containing *HaALS4* and *HaALS5* was observed on chromosome 5. The figure was generated using TBtools based on sunflower genome annotation files.

The 11 *HaALS* genes were unevenly localized across 10 of the sunflower chromosomes (Figure 1), with no ALS members detected on chromosomes 3, 8, 11, 12, 13, 14, and 17. *HaALS*1 was located at approximately 45 Mb on chromosome 1; *HaALS*2 was located at approximately 20 Mb on chromosome 2; *HaALS*3 was located at approximately 70 Mb on chromosome 4. A distinct gene cluster on chromosome 5 contained *HaALS*4 and *HaALS*5, located at approximately 140 Mb and 142 Mb, respectively, suggesting the possibility of a tandem duplication. *HaALS*6 was located at approximately 135 Mb on chromosome 6; *HaALS*7 was located at approximately 95 Mb on chromosome 7; *HaALS*8 was located at approximately 180 Mb on chromosome 9; *HaALS*9 was located at approximately 10 Mb near the centromere of chromosome 10; *HaALS*10 was located at approximately 5 Mb on chromosome 15; *HaALS*11 was located at approximately 150 Mb on chromosome 16. This scattered pattern of gene loci, along with the distinct gene cluster on chromosome 5, suggests that formation of the *HaALS* family was influenced by both tandem duplication and segmental duplication events.

### 2.3. Phylogenetic Relationships, Gene Structures, and Conservative Motifs of HaALS Genes

**Figure 2:**
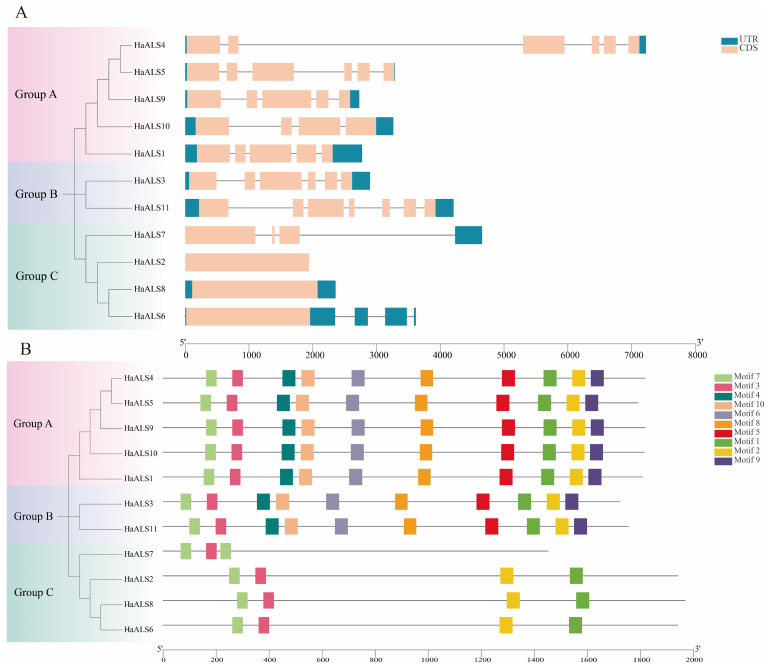
Phylogenetic relationships, gene structures, and conserved motifs of *HaALS* genes in sunflowers. (**A**) Gene structure. Dark blue: non-coding regions; colored boxes: coding sequences; lines: introns. (**B**) Conserved motifs. The scale indicates amino acid positions.

A rootless phylogenetic tree based on 11 HaALS protein sequences was constructed using the neighbor-joining (NJ) method, with 1000 bootstrap repetitions. The *HaALS* genes were classified into three evolutionary branches: Group A (*HaALS*4, *HaALS*5, *HaALS*9, *HaALS*10, *HaALS*1), Group B (*HaALS*3, *HaALS*11, *HaALS*7), and Group C (*HaALS*2, *HaALS*8, *HaALS*6). Each group is represented by a different background color in the figure (Figure 2).

Gene structures were determined by comparing genomic sequences and coding sequences (CDS) and visualizing them using GSDS 2.0 or TBtools. Untranslated regions (UTR) are indicated by dark blue boxes, CDS by colored boxes, and introns by connecting lines. Members of Group A typically possess multiple exons and introns, with relatively short exons and shorter 5′/3′ UTRs. Members of Group B (*HaALS*3, *HaALS*11, *HaALS*7) exhibited gene structures with multiple exons, some of which were notably longer; *HaALS*7 had fewer but longer exon-intron units. Group C members (*HaALS*2, *HaALS*8, *HaALS*6) had relatively simple gene structures; *HaALS*2 and *HaALS*8 possessed large, nearly continuous CDS regions with few introns, while *HaALS*6 had a more fragmented gene structure but remained distinctive. Gene structures within each phylogenetic group were generally consistent [19].

Conservative motif analysis using MEME identified 10 motifs. Group A members possessed a highly conserved motif sequence: motifs 7, 3, 4, 10, 6, 8, 5, 1, 2, and 9. Group B shared the same set of motifs but with different spacing or arrangement; the motif pattern of *HaALS*7 was slightly truncated. The motif composition of Group C was relatively simple, primarily limited to motifs 7, 3, and either 5 or 1. Many motifs present in Groups A and B were absent in Group C, suggesting that Group C has followed a unique evolutionary trajectory and may possess specialized functions.

### 2.4. Protein Domain Architecture and Sequence Conservation of HaALS Proteins

**Figure 3:**
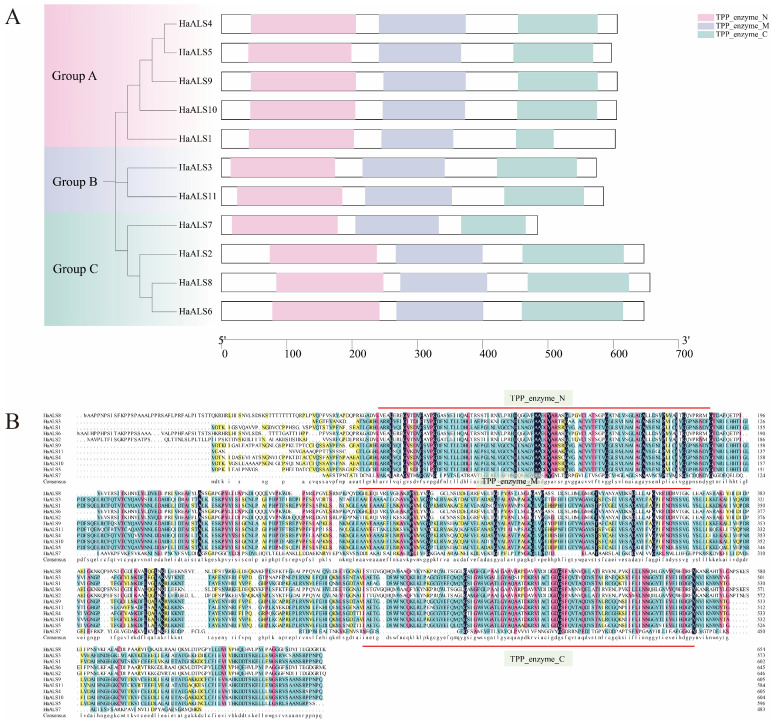
Domain architecture and sequence conservation of *HaALS* proteins. (**A**) Gene conserved domain architecture. The three characteristic TPP_enzyme domains. (**B**) Multiple sequence alignment of HaALS proteins. Red lines above the alignment indicate major conserved regions that are critical for ALS catalytic function. Domains are represented by different colors; the red lines above the alignment indicate major conserved regions.

Domain predictions (Pfam/SMART) and multiple sequence alignments indicate that all 11 HaALS proteins contain three characteristic TPP_enzyme (thiamine pyrophosphate-dependent enzyme) domains: TPP_enzyme_N (pink), TPP_enzyme_M (light blue), and TPP_enzyme_C (light green), which share similar relative positions in the sequence (Figure 3). The TPP_enzyme_N domain spans approximately residues 100–250, the TPP_enzyme_M domain spans approximately residues 300–550, and the TPP_enzyme_C domain spans approximately residues 550–700. The domain boundaries coincide with highly conserved regions critical for ALS function. The N-terminal and linker regions exhibit greater variability. Protein lengths vary (e.g., *HaALS*2 is one of the longest; *HaALS*3, *HaALS*7, and *HaALS*11 are relatively short), and the lengths of the linker regions between domains also differ among members. The conserved domain architecture supports the idea that these regions share a common catalytic mechanism and are subject to strong purifying selection.

### 2.5. Phylogenetic Analysis of HaALS Gene Promoters and Distribution of Cis-Acting Elements

**Figure 4:**
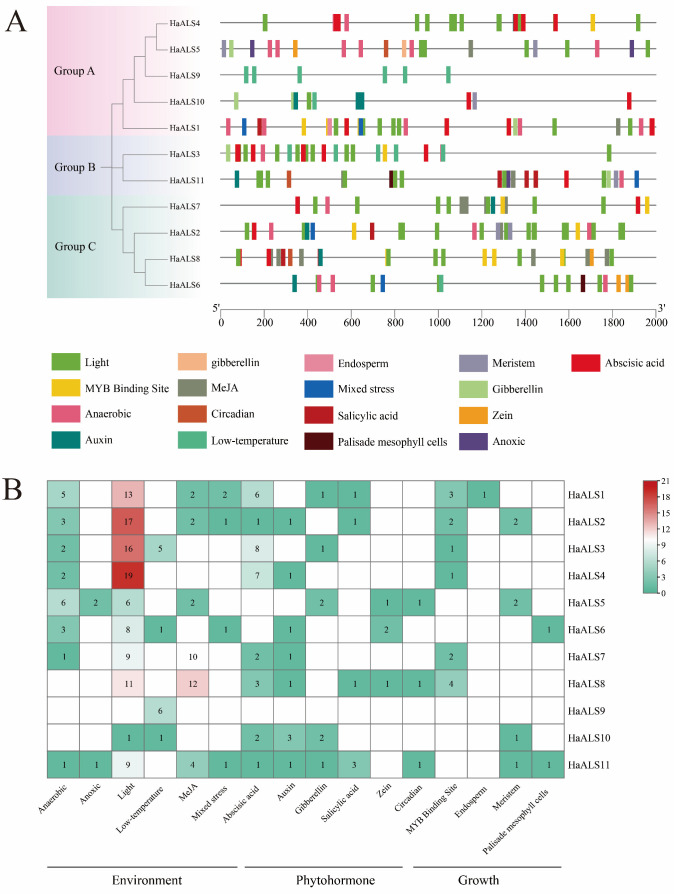
Phylogenetic tree and distribution of cis-acting elements in *HaALS* gene promoters. (**A**) On the left, a phylogenetic tree with three groups; Right: 2000 bp promoter region, with elements represented by colored boxes (see legend). (**B**) Heatmap of the number of cis-acting elements in *HaALS* gene promoters. Rows: genes; columns: element categories; color scale: copy number.

A phylogenetic analysis was performed again on the same 11 HaALS proteins, grouping them into three evolutionary branches: Group A (*HaALS*4, *HaALS*5, *HaALS*9, *HaALS*10), Group B (*HaALS*1, *HaALS*3, *HaALS*11), and Group C (*HaALS*2, *HaALS*6, *HaALS*7, *HaALS*8). The promoter region upstream of the translation start site of each *HaALS* gene (2000 bp) was extracted and analyzed using PlantCARE (Figure 4). Multiple cis-acting elements were detected, including: environmental (light response, anaerobic response, cold response, hypoxic response, mixed stress response); plant hormones (gibberellin response, methyl jasmonate response, auxin response, abscisic acid response, salicylic acid response); growth/development (endosperm expression, meristem expression, maize alcohol-soluble protein expression, palisade tissue cell expression, circadian rhythm regulation); and MYB-binding sites. The density and composition of elements varied among different genes: for example, *HaALS*1 and *HaALS*3 exhibited higher element density and diversity, whereas *HaALS*4 and *HaALS*5 showed a more concentrated or specific distribution of elements, suggesting that *HaALS* members possess distinct regulatory potentials [19].

The number of each type of cis-acting element in each gene was summarized and presented as a heatmap (Figure 4B), with elements classified into three categories: environmental, plant hormones, and growth. Environment: Light-responsive elements were the most abundant; *HaALS*4 (19), *HaALS*3 (17), *HaALS*2 (16), and *HaALS*1 (13) had the highest numbers. *HaALS*11 contained 11 hypoxia-responsive elements; *HaALS*8 contained 12 cold-responsive elements. Plant hormones: Abscisic acid response elements were abundant in *HaALS*4 (8), *HaALS*7 (7), and *HaALS*8 (7). Auxin response elements were relatively common, with *HaALS*8 containing 4. Methyl jasmonate response elements were particularly prominent, with 12 found in *HaALS*8. Growth: MYB-binding sites were frequently observed (e.g., 5 in *HaALS*1). Zea mays alcohol-soluble proteins, endosperm, meristem, palisade tissue, and circadian regulatory elements were also detected. The heatmap reveals a complex, gene-specific regulatory landscape, indicating that *HaALS* genes are regulated by a combination of light, hormonal, and developmental signals [19].

### 2.6. Phylogenetic Relationships of the ALS Gene Family in Sunflower, Lettuce, and Arabidopsis

**Figure 5:**
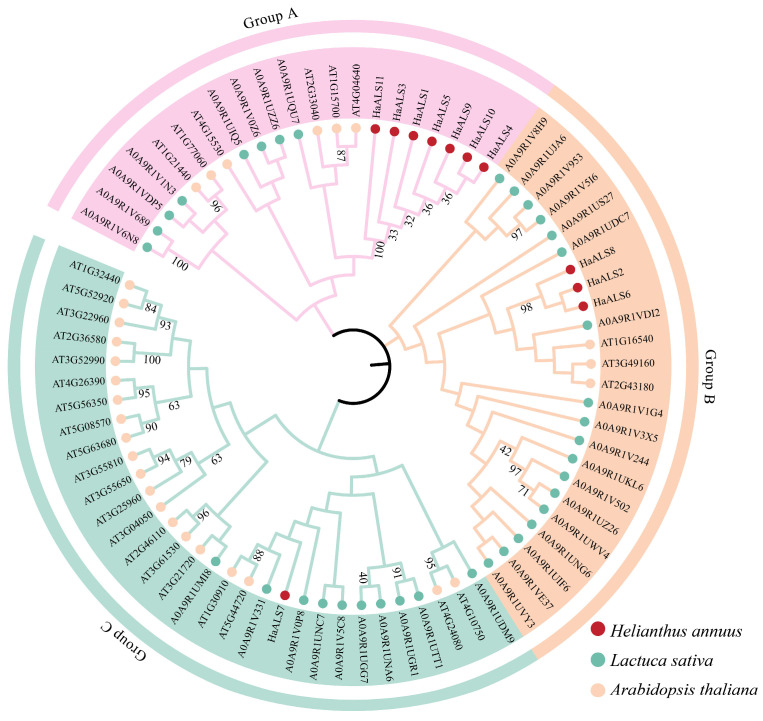
Phylogenetic tree of the *ALS* gene family in *Helianthus annuus*, *Lactuca sativa*, and *Arabidopsis thaliana*. Adjacency tree, 1000 bootstrap repetitions; bootstrap values > 30 are displayed at nodes. Red, sunflower (*Helianthus annuus*); blue-green, lettuce (*Lactuca sativa*); magenta, Arabidopsis (*Arabidopsis thaliana*).

A phylogenetic tree was constructed using the agglomerative method based on 11 *HaALS*, 29 lettuce (*Lactuca sativa*), and 25 Arabidopsis (Arabidopsis thaliana) ALS protein sequences, and 1000 bootstrap repetitions were performed (Figure 5). The ALS proteins were divided into three evolutionary branches (Group A, pink; Group B, magenta; Group C, cyan). Group A comprised seven *HaALS* genes (*HaALS*1, *HaALS*3, *HaALS*4, *HaALS*5, *HaALS*9, *HaALS*10, *HaALS*11) as well as homologous genes from lettuce and Arabidopsis (e.g., *A0A9R1V0Z6*, *A0A9R1UIQ5*; *AT1G15700*, *AT4G04640*). Group B comprised three *HaALS* genes (*HaALS*2, *HaALS*6, *HaALS*8) and homologs from both plants (e.g., A0A9R1V8H9, A0A9R1V953; AT1G16540, AT3G49160). Group C contained one *HaALS* gene, *HaALS*7, which clusters with numerous lettuce and Arabidopsis sequences (e.g., *A0A9R1UMI8*, *A0A9R1V0P8*; *AT1G32440*, *AT5G52920*). Key nodes exhibited high bootstrap support (e.g., 96–100%). The mixed distribution of the three species along evolutionary branches suggests that these ALS lineages have ancient origins and have undergone species-specific expansions or losses. In the figure, sunflower genes are marked with red circles, lettuce genes with blue-green circles, and Arabidopsis genes with magenta circles.

### 2.7. Chromosomal Distribution and Intragenomic Collinearity of Haals Genes

**Figure 6:**
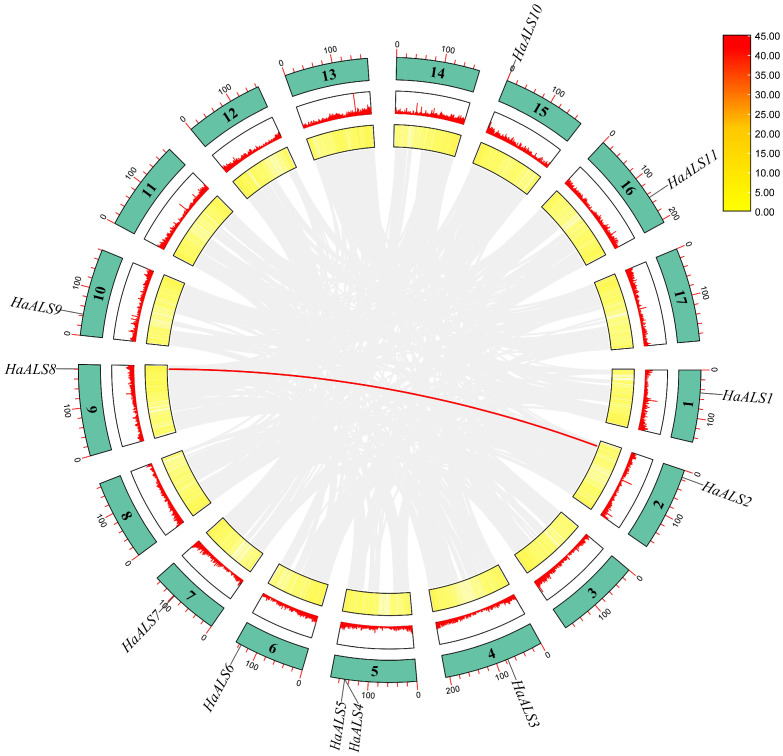
Chromosomal distribution and intragenomic collinearity of *HaALS* genes (Circos plot). Outer ring: 17 chromosomes; inner ring: gene density (yellow heatmap, red histogram); gray bands: collinearity blocks; red band: chromosome 8–chromosome 1 collinearity.

A Circos plot was used to illustrate the distribution of *HaALS* genes across the 17 chromosomes of sunflower and their intragenomic collinearity (Figure 6). The outer ring represents the 17 chromosomes and includes a scale (Mb). The two inner rings on each chromosome display *HaALS* gene density: the innermost ring is a yellow heatmap, and the adjacent ring is a red histogram; the color scale quantifies gene density from 0.00 to 45.00. The distribution of *HaALS* genes is uneven; several chromosomes (e.g., 1, 6, 9, 11, 12, 13, 14, 15, 16, 17) exhibit regions of higher gene density (distinct red peaks). The central region illustrates genomic collinearity: gray bands connect homologous regions, reflecting segmental duplications involving *HaALS* genes. The red band highlights a major collinearity block between chromosome 8 (*HaALS*8) and chromosome 1 (*HaALS*1), suggesting a significant duplication event or the presence of a highly conserved gene block. Taken together, this pattern indicates that segmental duplications, as well as possible whole-genome duplication events, have driven the expansion and distribution of the *HaALS* family in sunflowers.

### 2.8. Analysis of Interspecific Collinearity of ALS Genes Among Lettuce, Sunflower, and Arabidopsis

**Figure 7:**
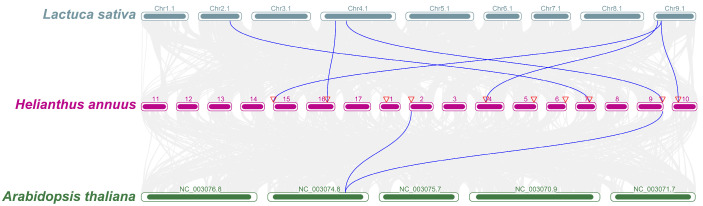
Interspecific collinearity of ALS genes among *Lactuca sativa*, *Helianthus annuus*, and *Arabidopsis thaliana.* Gray lines: background collinearity; blue curves: ALS gene collinearity; red triangles, *HaALS* loci.

An analysis of the interspecific collinearity of ALS genes was conducted for *Lactuca sativa* (upper ring, gray; chromosomes Chr1.1–Chr9.1), *Helianthus annuus* (middle ring, magenta; segments 1–17), and Arabidopsis thaliana (lower ring, dark green; NC_003076.8, NC_003074.8, NC_003075.7, NC_003070.9, NC_003071.7) (Figure 7). Thin gray lines indicate background collinearity blocks; thick blue curves connect homologous/paralogous ALS gene regions. Red inverted triangles on the sunflower segments mark the positions of *HaALS* genes. Multiple blue curves link sunflower segments (e.g., 15, 16, 1, 2, 4, 5, 6, 7, 9, 10) with lettuce (e.g., Chr3.1, Chr4.1, Chr8.1, Chr9.1) and Arabidopsis (e.g., NC_003074.8, NC_003075.7, NC_003070.9, NC_003071.7). Some sunflower regions (e.g., fragment 4) are linked to multiple chromosomes in lettuce and Arabidopsis, which is consistent with the duplication and conservative evolution of the ALS family in these dicotyledons.

### 2.9. Analysis of Evolutionary Selection Pressure (Ka/Ks) on HaALS Gene Pairs

**Figure 8:**
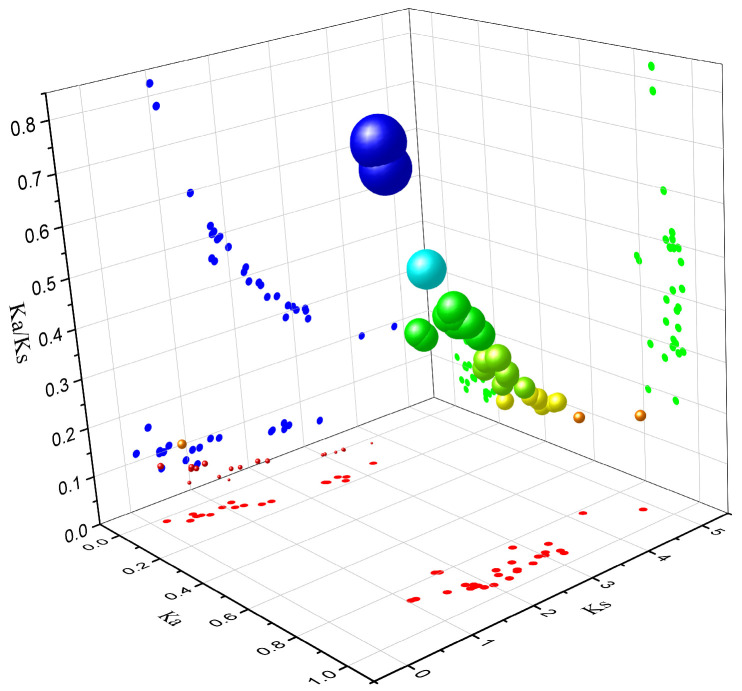
Analysis of evolutionary selection pressure (Ka/Ks) for *HaALS* gene pairs. 3D plot: *X*-axis, Ka; *Z*-axis, Ks; *Y*-axis, Ka/Ks. Colored spheres represent gene pairs; most points had Ka/Ks < 1.

The synonymous (Ks) and nonsynonymous (Ka) substitution rates, as well as the Ka/Ks ratios, were calculated for paralogous *HaALS* gene pairs and visualized using a three-dimensional scatter plot (Ka, Ks, Ka/Ks) (Figure 8). For the vast majority of gene pairs, Ka/Ks < 1, with values mostly ranging from 0 to approximately 0.85, indicating that the HaALS family has evolved primarily under purifying selection, wherein Ka/Ks < 1, Ka/Ks = 1, and Ka/Ks > 1 indicate purifying selection, neutral evolution, and positive selection, respectively [20]. A dense cluster of gene pairs (red spheres) exhibited very low Ka/Ks (e.g., <0.2) and low Ka values, consistent with recent duplication events and/or strong constraints. A second cluster of gene pairs (blue spheres) exhibited higher Ka/Ks values (approximately 0.4–0.85), though still <1, suggesting a relaxation of purifying selection or the presence of local variation. Ks values ranged from near 0 to over 5, indicating that duplication events occurred during different evolutionary periods. No gene pairs had Ka/Ks > 1; there is no evidence of genome-wide positive selection. Overall, purifying selection has maintained the conserved structure and function of the *HaALS* family in sunflowers.

### 2.10. Distribution of Transcription Factor Families and Regulatory Networks of HaALS Genes

**Figure 9:**
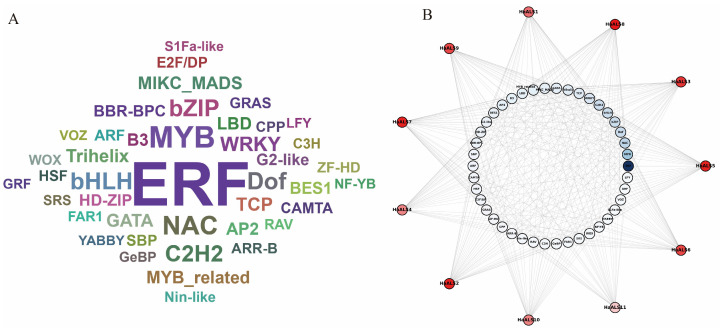
Transcription factor distribution and regulatory network of *HaALS* genes. (**A**) Word cloud of transcription factor family abundances in sunflowers. (**B**) Regulatory network: outer ring, *HaALS* genes (red/pink); center, transcription factor families (blue); edges, predicted regulatory interactions.

**Figure 10:**
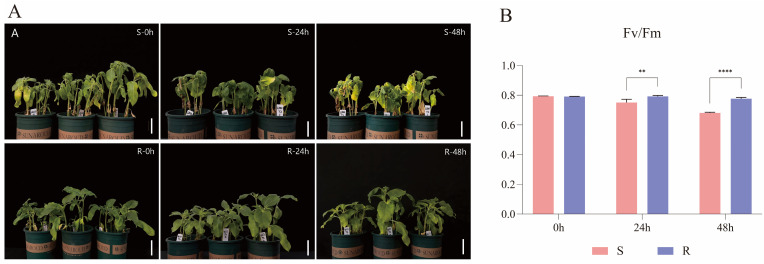
Morphological and physiological characterization of sunflower genotypes under imazethapyr stress. (**A**) Phenotypic comparison of susceptible (S) and resistant (R) plants at 0, 24, and 48 h post-treatment, showing severe wilting and chlorosis in the S genotype. (**B**) Changes in the maximum photochemical efficiency of PSII (Fv/Fm). Data represent the mean ± SD of three biological replicates. Asterisks indicate significant differences between R and S genotypes at each time point. ** *p* ≤ 0.01; **** *p* ≤ 0.001; one-way analysis of variance followed by Tukey’s honestly significant difference test.

Transcription factor (TF) families in sunflowers were identified, and their relative abundances were visualized as a word cloud (Figure 9A, Table S1). The ERF (ethylene response factor) family was the most abundant (largest font size). Other abundant families included MYB, bHLH, NAC, C2H2, bZIP, and MIKC_MADS; families such as GRAS, WRKY, HD-ZIP, ARF, B3, HSF, Dof, TCP, AP2, GATA, YABBY, SBP, VOZ, WOX, and Trihelix were also present. A regulatory network linking *HaALS* genes to transcription factor families was constructed (Figure 10B). The *HaALS* genes (*HaALS*1–*HaALS*11) formed the outer ring (red/pink nodes) and were extensively connected to a cluster of transcription factor family nodes (blue) at the center. Each *HaALS* gene was connected to multiple transcription factor families, suggesting the presence of combinatorial transcriptional regulation. The MIKC_MADS nodes were darker in color, indicating higher connectivity or centrality. This network indicates that *HaALS* gene expression is regulated by a wide range of transcription factors, with ERF, MYB, and MIKC_MADS likely playing particularly important roles.

### 2.11. Phenotypic and Photosynthetic Responses to Imazethapyr Stress

To evaluate the biological impact of imazethapyr, we monitored the morphological changes and photosynthetic efficiency of both resistant (R) and susceptible (S) sunflower genotypes (Figure 10). Under control conditions (0 h), both genotypes exhibited vigorous growth with healthy green leaves and comparable Fv/Fm values, recorded at 0.796 for the S genotype and 0.793 for the R genotype, respectively.

Following imazethapyr application, the S genotype displayed rapid and severe phenotypic deterioration. By 24 h, initial symptoms of leaf wilting and growth retardation were observed in S plants, correlating with a significant drop in Fv/Fm to 0.757. By 48 h, the S genotype exhibited characteristic herbicide injury symptoms, including intense chlorosis (yellowing) of the shoot apical meristem and severe epinasty of the primary leaves; these morphological symptoms were mirrored by a further decline in Fv/Fm to 0.686, indicating an impairment of the PSII reaction centers.

In contrast, the R genotype demonstrated remarkable tolerance throughout the treatment period. No visible symptoms of chlorosis or wilting were observed in R plants, which remained upright and green (Figure 10A). Consistent with this stable phenotype, the Fv/Fm values in the R genotype remained above 0.78 at 48 h, showing no significant deviation from the baseline (*p* > 0.05). These results indicate that the R genotype possesses a robust physiological defense mechanism that prevents both the biochemical inhibition of photosynthesis and the subsequent morphological damage triggered by imazethapyr.

### 2.12. Herbicide-Induced Expression Patterns of the HaALS Gene Family

**Figure 11:**
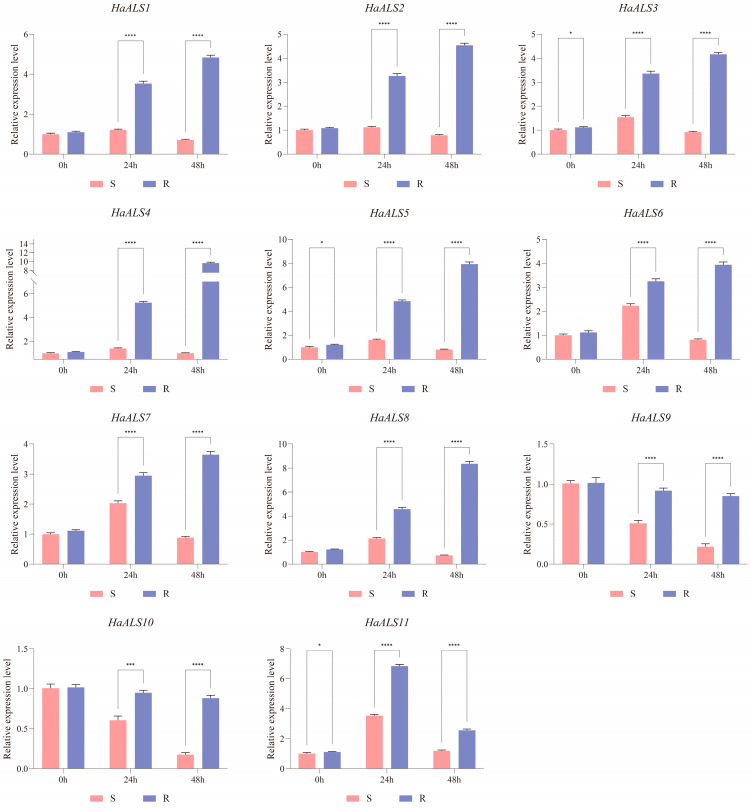
Relative expression profiles of the *Helianthus annuus* acetolactate synthase (*HaALS*) gene family in the leaves of imidazolinone-susceptible and imidazolinone-resistant sunflower cultivars following the foliar application of imazethapyr. Seedlings at the four-true-leaf stage were sampled at 0, 24, and 48 h after herbicide application, and transcript levels of individual HaALS genes were determined by quantitative real-time-polymerase chain reaction. Data represent mean ± standard error of three biological replicates. Asterisks indicate significant differences between susceptible and resistant genotypes at the same time point (* *p* ≤ 0.05; *** *p* ≤ 0.001; **** *p* ≤ 0.0001; one-way analysis of variance followed by Tukey’s honestly significant difference test).

To elucidate the transcriptional responses of the *HaALS* gene family to imidazolinone herbicide stress, the relative expression levels of eleven *HaALS* genes were quantified in leaves of the susceptible (S) and resistant (R) cultivars at 0, 24, and 48 h following the foliar application of imazethapyr. Overall, members of the *HaALS* family displayed pronounced herbicide-responsive expression patterns, with clear temporal and genotypic differentiation (Figure 11). In the absence of treatment (0 h), most *HaALS* genes showed comparable and relatively low transcript abundances between S and R plants. Upon herbicide exposure, however, the R genotype exhibited a rapid and sustained transcriptional activation of the majority of *HaALS* genes, whereas the S genotype showed only modest changes or even slight repression over the same time course, indicating a stronger and more coordinated transcriptional response in the resistant background.

Notably, *HaALS*1–*HaALS*3, *HaALS*4, *HaALS*5, and *HaALS*7–*HaALS*9 were markedly induced in the R genotype, with transcript levels increasing significantly at 24 h and reaching maxima at 48 h, with *HaALS4* reaching 6-fold at 24 h and >10-fold at 48 h, *HaALS11* reaching 6.6-fold at 24 h, and several other paralogs showing intermediate induction, relative to the untreated control. Among these, *HaALS4* displayed the most robust and sustained induction, with sharp increases already evident at 24 h and further amplification to >10-fold by 48 h, whereas HaALS11 reached its peak induction (6.4 fold) at 24 h and subsequently declined, yet remained above the baseline at 48 h, suggesting that they may function as primary contributors to the imidazolinone-resistance mechanism. In contrast, the expression of these genes in the S genotype remained relatively stable or exhibited only slight, transient fluctuations, resulting in highly significant expression differences between S and R at later time points. *HaALS*10 showed comparatively minor temporal variation in both genotypes, implying a more constitutive role with limited involvement in the herbicide-induced response. Collectively, these findings indicate that the enhanced and sustained induction of multiple *HaALS* members—particularly *HaALS*4 and *HaALS*11—in the resistant cultivar is closely associated with imazethapyr tolerance and likely constitutes a key component of the molecular basis underlying herbicide resistance in sunflower.

## 3. Discussion

Acetolactate synthase (ALS; EC 2.2.1.6) catalyzes the first key step in the biosynthesis of branched-chain amino acids (BCAAs), and is thus crucial for plant growth and nitrogen metabolism [1]. This central metabolic role explains why ALS has repeatedly been targeted by herbicides and why the selection pressure of ALS inhibitors has rapidly led to resistance in agricultural systems [21]. Imidazolinones, including imazethapyr and imazamox, are widely used Group 2 ALS inhibitors that provide broad-spectrum weed control and have been applied to a variety of crops and cultivation systems; in sunflower, the availability of imidazolinone-tolerant germplasm has further increased the reliance on this mode of action [22]. At the mechanistic level, resistance is usually attributed to amino acid substitutions at the target site, which reduce herbicide binding while maintaining catalytic activity, and recurrent “hotspot” sites (Pro197, Ala122, Trp574, Ser653 and related sites in the standardized ALS numbering scheme) are observed in different species [23]. However, focusing solely on single-site amino acid substitutions may obscure two increasingly important features in the crop genome: (i) ALS typically exists as a small gene family rather than a single copy; (ii) the phenotypic outcome under herbicide pressure is shaped by both the biochemical properties of the target enzyme and the regulatory state that determines the effective enzyme abundance. Against this background, this study provides a perspective at the ALS family level by integrating the evolutionary characteristics of the HaALS paralogous genes in sunflower with the genotype-specific transcriptional responses to imazethapyr in susceptible and resistant cultivars.

At the genomic level, we identified 11 *HaALS* genes distributed across the sunflower chromosomes. These widely dispersed loci, along with local obvious clustering phenomena, are consistent with the expansion of the gene family driven by tandem and/or segmental duplication. Phylogenetic reconstruction divided the *HaALS* proteins into three branches (Groups A-C), and the exon–intron structure and composition of conserved motifs further supported the branch-dependent differentiation. Importantly, all *HaALS* proteins retained the three characteristic thiamine pyrophosphatase enzyme domains (TPP_enzyme_N, TPP_enzyme_M, and TPP_enzyme_C), and their positions were highly conserved, which strengthened the view that the ALS catalytic structure was preserved during the evolution of sunflower.

Consistent with the biochemical necessity of ALS activity, selective analysis indicated that most *HaALS* paralogous gene pairs evolved under purifying selection (Ka/Ks < 1), suggesting a strong constraint on amino acid change-type differentiation. This evolutionary outcome is instructive under herbicide pressure: sunflowers may mainly achieve adaptive flexibility through regulatory differentiation (such as cis-element variation and genotype-specific expression programs) rather than extensive protein innovation among paralogous genes, while maintaining a stable catalytic core [24]. A similar pattern has been described in other key metabolic enzymes subject to strict structural constraints, where the retention of paralogous genes is more likely explained by dosage effects, tissue specificity, or stimulus-responsive regulation, rather than the main catalytic neofunctionalization [25].

Supporting this regulatory differentiation framework is the fact that promoter analysis revealed a rich repertoire of cis-acting regulatory elements at the *HaALS* loci, encompassing environmental response motifs, growth/development-related signals, and hormone-related elements. Notably, ABA and methyl jasmonate (MeJA) response motifs often coexist with hypoxia/low oxygen response, light regulation, and complex stress signal-related elements. Although the cis-element library itself cannot predict inducibility, this promoter map provides mechanistic rationality for the differential transcriptional recruitment of specific *HaALS* paralogous genes under plant toxicity perturbations [24]. This regulatory plasticity is particularly important in the context of ALS inhibition, as herbicide exposure can trigger acute metabolic bottlenecks: the restriction of BCAA biosynthesis can constrain protein synthesis and growth, and disrupt the broader metabolic fluxes dependent on amino acid availability [26]. Therefore, the transcriptional regulation of ALS paralogous genes can be regarded not only as a passive response to stress, but also as a potential compensatory lever to buffer pathway output under chemical challenges [27].

Unlike the gene-by-gene description of the expression profile, our key inference from the time course of imazethapyr is that the resistant variety shows a stronger coordinated *HaALS* transcriptional response than the susceptible variety (Figure 11). Importantly, this differentiation is both qualitative and quantitative at the level of representative paralogous genes: in the resistant background, *HaALS*4 is significantly induced at 24 h, rising to about 6-fold, and exceeds about 10-fold at 48 h (relative to 0 h), while *HaALS*11 rises to about 6–7-fold at 24 h, then partially declines, but remains above the baseline at 48 h. Multiple other members (*HaALS*1/2/3/5/6/7/8) show moderate induction in the resistant variety (typically about 3–8-fold at 48 h), while the same genes in the susceptible variety are close to the baseline (typically about 0.8–2-fold) or show slight attenuation at later time points. These effect sizes support the view that herbicide tolerance is associated with genotype-specific capacity to amplify ALS family transcription within the post-application window, rather than being solely explained by constitutive expression differences [28].

This genotype-dependent regulatory capacity is consistent with two non-mutually exclusive frameworks emerging from ALS-related research. Firstly, in the context of carrying imidazolinone-insensitive ALS alleles (such as in many “Clearfield” type systems), the inducible transcription of other paralogous genes may enhance tolerance by increasing the total ALS protein abundance, thereby expanding the effective target pool and stabilizing the residual flux of BCAA biosynthesis [29]. In this scenario, target site insensitivity ensures catalytic function, while transcriptional compensation improves metabolic robustness under continuous inhibitor presence [30]. Secondly, transcriptional differentiation may reflect a broader regulatory reprogramming associated with herbicide tolerance, including differences in stress signaling and metabolic homeostasis that indirectly regulate ALS gene expression [31]. Notably, herbicide responses often encompass pleiotropic physiological adjustments (such as changes in growth dynamics, chloroplast function, and resource allocation), which can feedback on core metabolic genes [32].This explanation is consistent with reports that herbicide tolerance or resistance involves both target site and non-target site components, including enhanced detoxification, altered transport, and stress adaptation pathways, which collectively influence the apparent sensitivity of plants to herbicide challenges [33].

The uneven response among *HaALS* members further supports the existence of regulatory domains within the family. In other plant systems, the specific regulation of ALS paralogous genes is related to promoter structure and chromatin environment, leading to differential inducibility under exogenous or environmental cues [10]. This promoter-level specialization provides a reasonable explanation for why only some ALS paralogous genes exhibit strong induction modules after ALS inhibition, while others remain relatively stable. Importantly, this partitioning may have practical significance: inducible paralogous genes may contribute more to tolerance during the window period after application (when inhibitor pressure is the greatest), while constitutively expressed paralogous genes may maintain basal BCAA flux under non-stress conditions [34,35]. Additionally, as ALS targets the chloroplast in most species, regulatory responses that maintain chloroplast protein homeostasis and photosynthetic function may cross with ALS transcription, providing an additional layer for tolerant genotypes to maintain performance under ALS inhibition [36]. Multiple studies have linked ALS inhibition to chlorophyll loss and photosynthetic damage, while tolerant lines can show better maintenance of photosynthetic parameters—these effects may result from the combined action of resistant target enzymes and compensatory regulatory programs [37].

**Figure 12:**
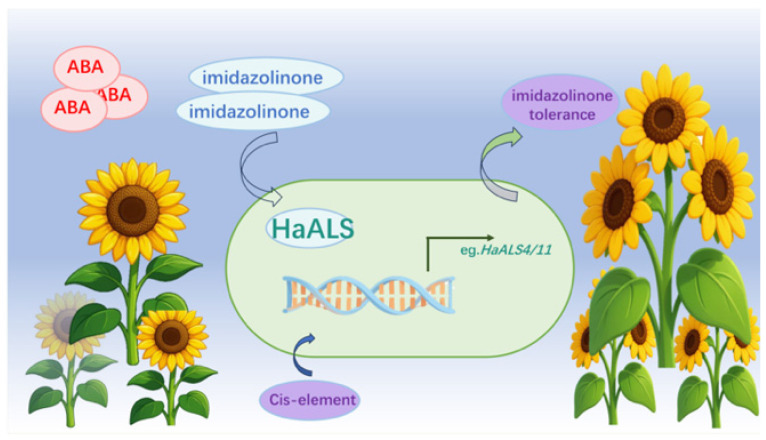
A working model of *HaALS* response to imidazolinone.

The physiological and phenotypic divergence observed between the resistant (R) and susceptible (S) genotypes provides robust evidence for the functional importance of the *HaALS* gene family in herbicide tolerance. In the S genotype, the significant decline in Fv/Fm (from 0.796 to 0.686) and the accompanying apical chlorosis under imazethapyr stress reflect a classic secondary effect of ALS inhibition. The depletion of branched-chain amino acids (BCAAs) following herbicide application likely restricts the de novo synthesis of the D1 protein, a core component of the PSII reaction center with a high turnover rate, thereby triggering irreversible photoinhibition and the breakdown of the photosynthetic apparatus [34,35]. In contrast, the R genotype exhibited remarkable physiological resilience, maintaining a healthy phenotype and stable Fv/Fm values (~0.79) throughout the 48 h treatment. This resilience is highly consistent with our transcriptional data, suggesting that the rapid and robust upregulation of *HaALS4* and *HaALS11* in the R genotype serves as a “transcriptional buffering” mechanism. By increasing the abundance of these key transcripts, R plants can potentially maintain sufficient ALS enzymatic activity to satisfy BCAA requirements, thereby shielding the photosynthetic machinery from metabolic starvation. These results bridge the gap between genomic characterization and practical herbicide tolerance, identifying specific *HaALS* paralogs as pivotal determinants of physiological adaptation in sunflower. The functions of *HaALS* genes need to be further investigated in depth, and Figure 12 shows a potential regulatory model of *HaALSs*.

## 4. Materials and Methods

### 4.1. Identification of Members of the HaALS Gene Family in Sunflower (Helianthus annuus L.)

The sunflower acetyl-L-lactate synthase (ALS) gene family was identified through homology searches based on query sequences, combined with the validation of conserved domains. Specifically, genomic sequences and protein annotation information for sunflower were obtained from public databases (such as NCBI/Phytozome or sunflower genome resources). Known ALS protein sequences from Arabidopsis were retrieved from TAIR (https://www.arabidopsis.org/, accessed 18 March 2026), used as query sequences, and BLASTP searches were performed against the sunflower protein database using the NCBI BLAST platform (https://blast.ncbi.nlm.nih.gov/Blast.cgi, accessed 18 March 2026). Candidate homologous sequences were retained based on an E-value threshold of 1 × 10^−5^ and/or 1 × 10^−10^, and redundant or incomplete sequences were excluded. The candidate sequences were submitted to Pfam and SMART to further validate the presence of ALS-associated conserved domains. The final members were named *HaALS*1 to *HaALS*11 based on their order on the sunflower chromosome. The physicochemical properties of *HaALS* proteins (amino acid length, molecular weight (Mw), theoretical isoelectric point (pI), instability index, hydrophobicity index, and average hydrophilicity (GRAVY)) were predicted using ExPASy ProtParam (https://web.expasy.org/protparam/, accessed 18 March 2026) and are summarized in Table 1.

### 4.2. Phylogenetic Analysis of HaALS Proteins

To further elucidate the evolutionary relationships between *HaALS* proteins and other plant ALS members, a multiple sequence alignment was performed based on the full-length *HaALS* sequence. Alignment results were generated using MUSCLE (or ClustalW/MAFFT), and a phylogenetic tree was constructed using the neighbor-joining (NJ) method in MEGA (MEGA X/MEGA 11) (Poisson model, with paired-deletion of gaps). Branch support was assessed through 1000 bootstrap repetitions. For cross-species comparisons, the same strategy was employed to obtain ALS protein sequences from lettuce and Arabidopsis, which were then included in the alignment and phylogenetic analysis. The final NJ tree was visualized and annotated using iTOL (https://itol.embl.de/, accessed 23 March 2026) or Evolview (https://www.evolgenius.info/, accessed 23 March 2026) to aid in the interpretation of phylogenetic relationships.

### 4.3. Chromosomal Localization Density and Gene Collinearity Analysis of HaALS Genes

Based on sunflower genome annotation data, TBtools (V2.080) was used to analyze the chromosomal distribution and gene density of *HaALS* family members. First, download relevant input data from the corresponding sunflower genome resources. To identify collinearity and orthologous gene arrangements, use MCScanX (V2.3.1) with all-vs-all BLASTP results and gene annotation files (GFF3/GTF) as inputs to detect and analyze collinearity blocks within the genome. Use Circos (or TBtools) to visualize genomic collinearity and the chromosomal distribution of *HaALS* genes, highlighting conserved genomic regions and potential duplication events.

### 4.4. Analysis of Conserved Domains and Gene Structures of HaALS Genes in Sunflower

To analyze the conserved domain architecture and gene structural features of *HaALS* family members, domains were annotated using the SMART online tool (https://smart.embl.de/, accessed 24 March 2026), and the NCBI Conserved Domain Database (CDD; https://www.ncbi.nlm.nih.gov/Structure/cdd/, accessed 24 March 2026). MEME Suite 5.x (https://meme-suite.org/, accessed 24 March 2026) was used to predict conserved motifs in *HaALS* protein sequences using default settings and a maximum motif count of 10. The predicted motifs were visualized in conjunction with gene structure maps (exon-intron organization), which were generated using GSDS 2.0 (http://gsds.gao-lab.org/ or http://gsds.cbi.pku.edu.cn/, accessed 24 March 2026) and/or TBtools based on GFF annotations.

### 4.5. Analysis of Cis-Regulatory Elements in HaALS Promoters

To identify potential cis-regulatory elements involved in *HaALS* transcriptional regulation, the 2000 bp sequence upstream of the translation start codon (ATG) of each *HaALS* gene was extracted as the promoter region. PlantCARE (https://bioinformatics.psb.ugent.be/webtools/plantcare/html/, accessed 18 March 2026) was used to analyze cis-acting elements within the promoter regions. TBtools (V2.080) was used to visualize the promoter structure and the distribution of cis-regulatory elements, and the cis-regulatory elements were further classified based on functional annotations (e.g., environmental response, plant hormone, and growth and development-related elements).

### 4.6. Plant Materials and Stress Treatments

Sunflower (*Helianthus annuus* L.) cultivars with contrasting herbicide tolerance—the imidazolinone-resistant ‘9077’ and the susceptible ‘LKZ12’—were employed in this study. Seeds were surface-sterilized in 1% (*v*/*v*) NaClO for 10 min, followed by five consecutive rinses with distilled water, and then germinated in plastic pots containing a sterilized peat–vermiculite mixture (1:1, *v*/*v*). Seedlings were maintained in a controlled environment chamber under a 16-h light/8-h dark photoperiod at 25 °C/20 °C, with a photosynthetic photon flux density of 450 μmol·m^−2^·s^−1^ and a relative humidity of 65%.

At the four-true-leaf stage (V4), uniform seedlings were subjected to herbicide stress. The treatment group received a foliar application of 5% imazethapyr aqueous solution administered at a standardized rate of 1500 mL/ha (corresponding to 75 g a.i./ha) using a precision manual sprayer until the foliage reached the point of incipient runoff.

At 0, 24, and 48 h post-application, the maximum photochemical efficiency of photosystem II (Fv/Fm) was first determined on the uppermost fully expanded leaves following a 30-min dark adaptation using a portable chlorophyll fluorometer. Immediately following the non-destructive fluorescence measurements, these identical leaves were harvested.

Each experimental unit comprised three biological replicates, with each replicate consisting of pooled tissue from three individual plants. All samples were immediately flash-frozen in liquid nitrogen and stored at −80 °C for subsequent total RNA extraction and transcriptional profiling of the *HaALS* gene family.

### 4.7. Gene Expression Analysis by qRT-PCR

Total RNA was extracted from the collected tissues using a plant RNA extraction kit (TIANGEN Biotech Co., Ltd., Beijing, China) according to the manufacturer’s instructions. RNA integrity and concentration were assessed via agarose gel electrophoresis and a Nanodrop 2000 spectrophotometer (A260/A280 ratio, Thermo Fisher Scientific, Waltham, MA, USA). Where necessary, DNase I treatment was performed to remove residual genomic DNA. Synthesize cDNA using 1 μg of total RNA and HiScript II Q Select RT SuperMix (for qRT-PCR, Vazyme, Nanjing, China) or an equivalent reverse transcription system. Perform qRT-PCR reactions in a 10-μL reaction volume on a real-time PCR system (CFX96 Touch, Bio-Rad, Hercules, CA, USA) using a SYBR Green-based master mix (ChamQ Universal SYBR qPCR Master Mix, Vazyme). Specific primers for the *HaALS* gene were designed using Primer Premier 5.0; the primer sequences are shown in Table S2. The qRT-PCR program consisted of an initial denaturation at 95 °C for 30 s, followed by 40 cycles of denaturation at 95 °C for 5 s and annealing/extension at 58 °C for 30 s. Melting curve analysis was performed using default settings to confirm amplification specificity. Relative expression levels were calculated using the 2^(−ΔΔCt) method, with three technical replicates per biological replicate. Gene expression was normalized to an internal reference gene (ACT2 or a housekeeping gene validated in this study). Upregulation was considered significant when relative expression was ≥2, and downregulation was considered significant when relative expression was ≤0.5; statistical significance was determined using Tukey’s HSD test (*p* < 0.05). Based on normalized expression values, expression profile heatmaps were generated using HemI 1.0 software.

### 4.8. Statistical Analysis

Differential expression between treatment and control groups, as well as between susceptible (S) and resistant (R) genotypes at each time point, was assessed using one-way analysis of variance (ANOVA) followed by Tukey’s honestly significant difference (HSD) test. Upregulation was considered significant when the relative expression was ≥2-fold, and downregulation was considered significant when the relative expression was ≤0.5-fold. Statistical significance was defined at *p* < 0.05. Data are presented as the mean ± standard error (SE) of three biological replicates.

## 5. Conclusions

In this study, we identified and characterized 11 *HaALS* genes in sunflower, including their chromosomal distribution, phylogenetic relationships, conserved domain architecture, evolutionary selection constraints, and promoter cis-regulatory features. Expression profiling by qRT-PCR under imazethapyr exposure demonstrated that *HaALS* genes undergo rapid and genotype-dependent transcriptional reprogramming. The resistant cultivar displayed a coordinated induction of multiple paralogs, with particularly strong and sustained responses of *HaALS*4 (>10-fold at 48 h) and *HaALS*11 (~6–7-fold at 24 h), whereas the susceptible cultivar exhibited comparatively weak transcriptional changes. These results support a model in which a conserved ALS catalytic core is complemented by regulatory divergence among paralogs, and nominate *HaALS*4 and *HaALS*11 (together with other inducible members) as priority candidates for dissecting the molecular basis of imidazolinone tolerance and for developing markers and strategies toward durable herbicide-tolerant sunflower germplasm.

## Figures and Tables

**Table 1:** Physicochemical properties of members of the sunflower acetyl-L-lactate synthase (*HaALS*) gene family.

Gene ID	Gene Name	Number of Amino Acid	Molecular Weight	Theoretical pI	Instability Index	Aliphatic Index	Grand Average of Hydropathicity	Prediction of Subcellular Localization
Chr01g0009771	*HaALS*8	1968	162,185.29	4.92	38.27	23.02	0.707	chlo
Chr02g0049991	*HaALS*3	1722	142,262.16	4.94	43.99	25.96	0.809	cyto
Chr04g0160511	*HaALS*1	1809	149,239.59	4.95	44.88	24.43	0.711	cyto
Chr05g0225421	*HaALS*6	1941	159,847.86	4.92	37.43	23.34	0.722	chlo
Chr05g0225471	*HaALS*2	1941	160,602.92	4.93	45.52	26.38	0.766	chlo
Chr06g0276351	*HaALS*9	1818	151,173.14	4.94	47	24.26	0.72	cyto
Chr07g0296351	*HaALS*11	1755	144,189.72	4.97	39.39	27.12	0.737	chlo
Chr09g0412081	*HaALS*4	1818	150,672.12	4.96	44.56	26.29	0.724	cyto
Chr10g0430561	*HaALS*10	1815	150,191.42	4.94	49.04	25.56	0.764	cyto
Chr15g0671131	*HaALS*5	1791	148,599.16	4.95	47.23	26.47	0.745	cyto
Chr16g0757621	*HaALS*7	1452	119,218.56	5	33.76	23.76	0.672	cyto

## Data Availability

All data are included in the main text.

## Supplementary Materials

The following supporting information can be downloaded at: https://www.mdpi.com/article/10.3390/plants15142113/s1: Table S1: Transcription factor prediction; Table S2: Primer sequences used for qRT-PCR analysis of *HaALS* genes in sunflower.
